# When Shall Gonorrhœries Marry?

**Published:** 1896-08

**Authors:** 


### Editorial.

### WHEN MAY GONORRHŒICS MARRY?

Lowenhardt (*Journal des Connaissances Medicals*) gives rules to be observed by physicians consulted by blenorrhagics to gain medical consent to marry.

Since the virulence of the urethral discharge intimately depends upon the presence of the gonococcus, the candidate for marriage should be subjected to numerous bacteriological examinations, carried out separately on the secretion of the anterior and the posterior urethra. A slight secretion is not sufficient for this purpose, but the urethral unicosa must be irritated in such a manner as to place it in analogous conditions to those (excess in *Baccho et Venere*), which light up an indolent process. The best means of obtaining this result is by injecting a few drops of a five per cent. solution of silver nitrate into the urethra; if then the discharge thus set up contains no gonococci, but is entirely made up of masses of epithelial cells, marriage can be permitted. The presence of numerous pus corpuscles necessitates renewed examinations and energetic treatment of this pseudo-gonorrhœa.

Lowenhardt insists upon the recognition of the fact that the gonococcus is alone responsible for the virulence of the exudate, and the serious complications observed in the genital apparatus of women.

Another rather popular method of provoking a urethral discharge in order to establish the verity of a cure, is to give an injection of a $\frac{1}{1000}$ bichloride solution, and to instruct the patient to drink a quart or more of beer. This would seem to be more heroic than the circumstances warrant. In spite of failure to find gonococci in the urethral discharge after repeated examinations, it is better to wait until the discharge has ceased entirely, and to withhold consent to marry until there can be no peradventure of contagion.

The extreme views of Nœggerath and Tait on the incurability of gonorrhœa in the male are too often and too clearly refuted by practical experience to merit serious consideration. Latent gonorrhœa in the etymological restriction of the adjective to lying hid, has no existence. If the disease exists, it can always be discovered. This fact removes that bugbear over which so many gynecologists have worked themselves into a fine frenzy.

